# Cronkhite—Canada syndrome associated with perianal condyloma acuminatum with malignant transformation: A case report

**DOI:** 10.1097/MD.0000000000025067

**Published:** 2021-03-12

**Authors:** Wei Wang, Xian-yong Cheng, Feng Xue, Hai-yan Liu, Hai-feng Lian, Cheng-xia Liu

**Affiliations:** Binzhou Medical University Hospital, The Yellow Rive Second Road, Binzhou City, Shandong Province, China.

**Keywords:** condyloma acuminatum, Cronkhite—Canada syndrome, diarrhea, gastrointestinal polyposis

## Abstract

**Rationale:**

Cronkhite-Canada syndrome (CCS) is a rare non-familial polyposis syndrome characterized by multiple gastrointestinal polyps with the ectodermal triad. To date, many complications of CCS have been reported in the literature, but perianal condyloma acuminatum with malignant transformation has not been included.

**Patient concerns:**

This report presents the case of a 52-year-old Chinese man who presented with diarrhea, loss of appetite, and weight loss. He developed skin pigmentation and atrophy of the fingernails and toenails. Upper gastrointestinal endoscopy, colonoscopy, capsule endoscopy, and enteroscopy revealed diffuse polyps along the entire digestive tract. Histopathological examination revealed polyps of different pathological types dominated by hamartoma. Physical examination revealed a crissum cauliflower-like neoplasm (2.5 × 2.0 cm). After perianal tumor resection, pathology suggested that this was a perianal condylomatous lesion with malignant transformation, as well as well-differentiated squamous cell carcinoma.

**Diagnoses:**

These clinical features and endoscopic findings were consistent with CCS which associated with perianal condyloma acuminatum with malignant transformation.

**Intervention:**

Clinical remission was achieved with glucocorticoid, azathioprine, and nutritional support.

**Outcome:**

At the 4-year follow-up, the patient had no diarrhea or loss of appetite, had gained 13 kg in weight, and the perianal tumor had not recurred.

**Lessons:**

No previous report has described CCS in a patient with perianal condyloma acuminatum with malignant transformation. As both conditions are related to immune disorders, their occurrence may be correlated.

## 1 Introduction

Cronkhite-Canada syndrome (CCS) is a rare non-inherited disease of unclear etiology characterized by gastrointestinal polyposis and an ectodermal triad including alopecia, cutaneous hyperpigmentation, and onychodystrophy.^[1]^ Patients with CCS may present with clinical symptoms, including chronic diarrhea, anorexia, ageusia, and emaciation. Various diseases have been reported to occur concomitantly with CCS. Here, we describe a case of CCS in a patient with perianal condyloma acuminatum with malignant transformation.

## 2 Case presentation

### 2.1 Chief complaints

A 52-year-old Chinese man presented with a history of diarrhea lasting 10 months (5–6 episodes per day, with liquid yellow stools). He gradually lost his appetite, felt sick when eating fatty foods, and had substantially reduced his food intake.

### 2.2 History of presenting illness

About 50 days after the onset of diarrhea, skin hyperpigmentation and atrophy of the fingernails and toenails began to appear. All the symptoms continuously increased. The findings of upper gastrointestinal endoscopy performed in the hospital suggested hypertrophic gastritis with extensive erosion. The patient demonstrated substantial weight loss (about 23 kg) since disease onset.

### 2.3 History of illness

The patient was previously healthy, and he had no family history of gastrointestinal polyposis. In the past half year, a cauliflower-like neoplasm was observed around the anus, of approximately 1.0 cm in size initially, which grew rapidly to more than 2 cm in the past 2 months.

### 2.4 Physical examination

**Figure 1:**
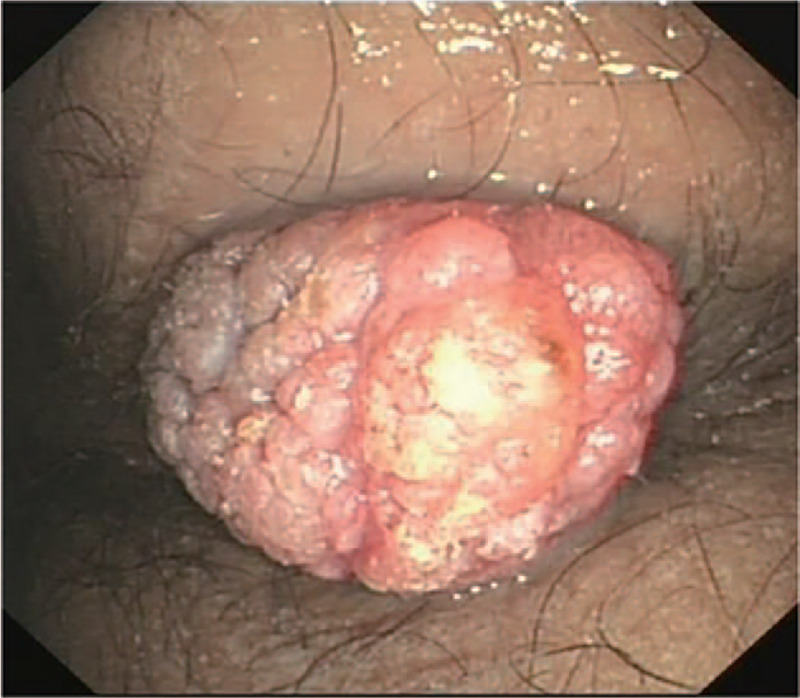
Perianal neoplasms as seen on physical examination.

The patient appeared emaciated. Physical examination findings included a sunken abdomen, systemic cutaneous hyperpigmentation (mainly on the hands, feet, and forehead), and apparent atrophy of the fingernails and toenails. A cauliflower-like bulge (2.5 × 2.0 cm) with a soft texture and congested surface was seen outside the anus (Fig. 1)

### 2.5 Laboratory examinations

Laboratory tests revealed that the patient's white blood cell count was 8.50 × 10^9^/L, hemoglobin was 160 g/L, and the platelet count was 343 × 10^9^/L. The fecal occult blood test was positive (++). Serum albumin was 27.8 g/L, and the globulin was 21.8 g/L. Antinuclear antibodies were all negative. The erythrocyte sedimentation rate, C-reactive protein, immunoglobulin, complement system, and other immunological indicators were all normal.

### 2.6 Imaging examinations

**Figure 2:**
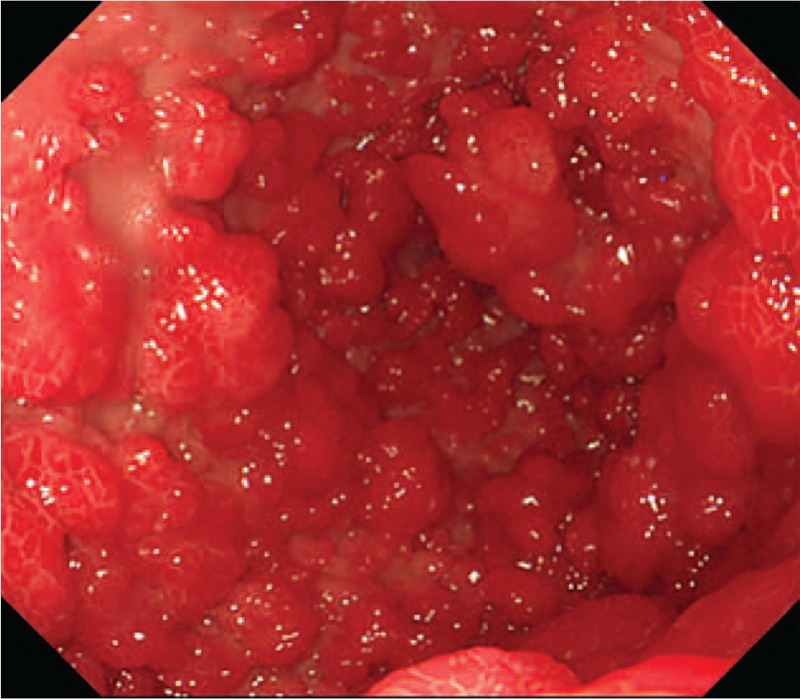
Upper gastrointestinal endoscopy: the polyps in the gastric antrum show a carpet-like distribution.

**Figure 3:**
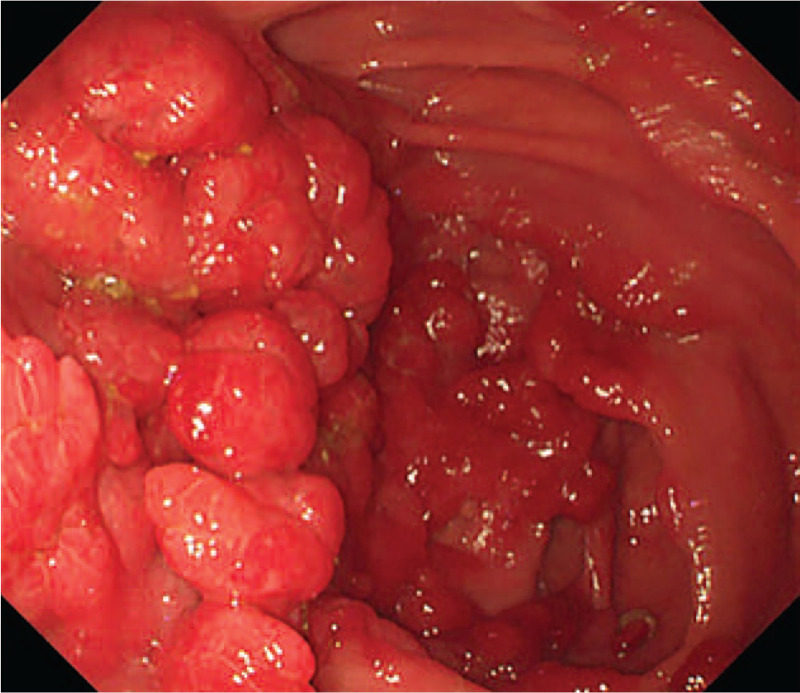
Colonoscopy: the polyps in the ileocecal junction are distributed in clusters.

**Figure 4:**
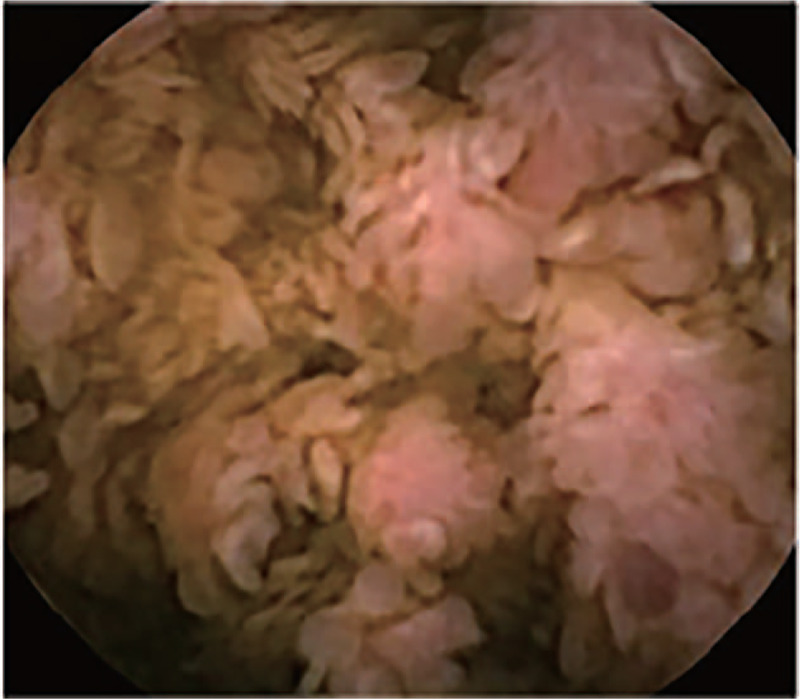
Capsule endoscopy: scattered polyps are seen in the jejunum.

**Figure 5:**
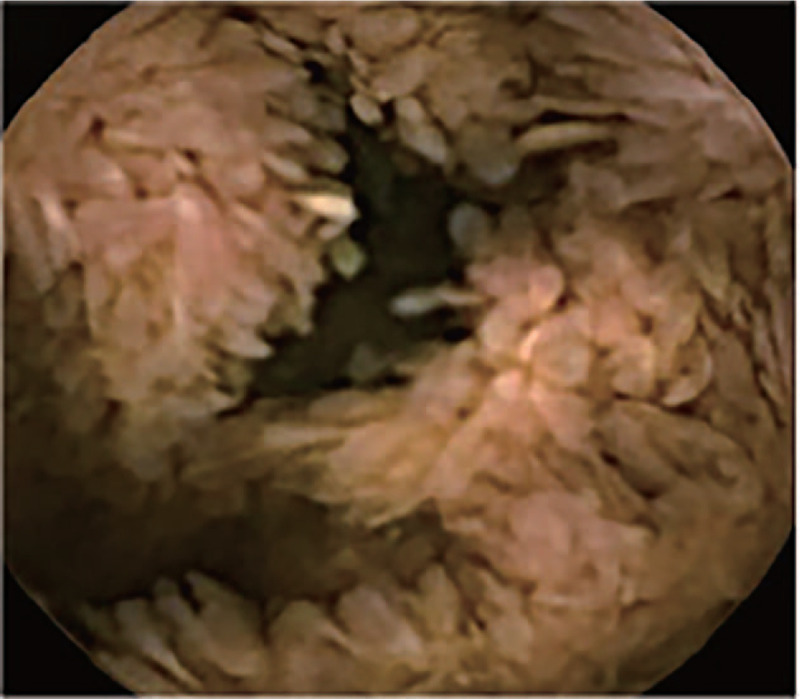
Capsule endoscopy: the intestinal villi are hyperplastic, prolonged, and demonstrate a seaweed-like pattern.

Abdominal computed tomography indicated uneven gastric wall thickening. Upper gastrointestinal endoscopy revealed that the esophageal mucosa was smooth without any polyps, but there were diffusely distributed 0.3-1.5-cm polyps in the gastric cavity. These were mainly flat polyps, mixed with some semi-pedunculated polyps, with significant hyperemia on the surface. The polyps in the antrum and gastric body showed a carpet-like distribution, and normal gastric mucosa was rarely seen (Fig. 2). Colonoscopy revealed hundreds of polyps (0.2–2.0 cm in diameter) scattered throughout the terminal ileum, colon, and rectum. Marked vascular congestion was found on the surface of these polyps. The polyps in the ileocecal junction and the proximal part of the ascending colon were distributed in clusters. The ileocecal valve was not normal-shaped and was covered by hundreds of polyps of different sizes (Fig. 3). Single balloon enteroscopy via the transoral and transanal approaches showed scattered polyps (0.3–1.2 cm in diameter), which were more densely distributed in the duodenum, proximal jejunum, and terminal ileum. Capsule endoscopy magnification revealed that the intestinal villi were hyperplastic, prolonged, and presenting a seaweed-like appearance (Figs. 4 and 5).

**Figure 6:**
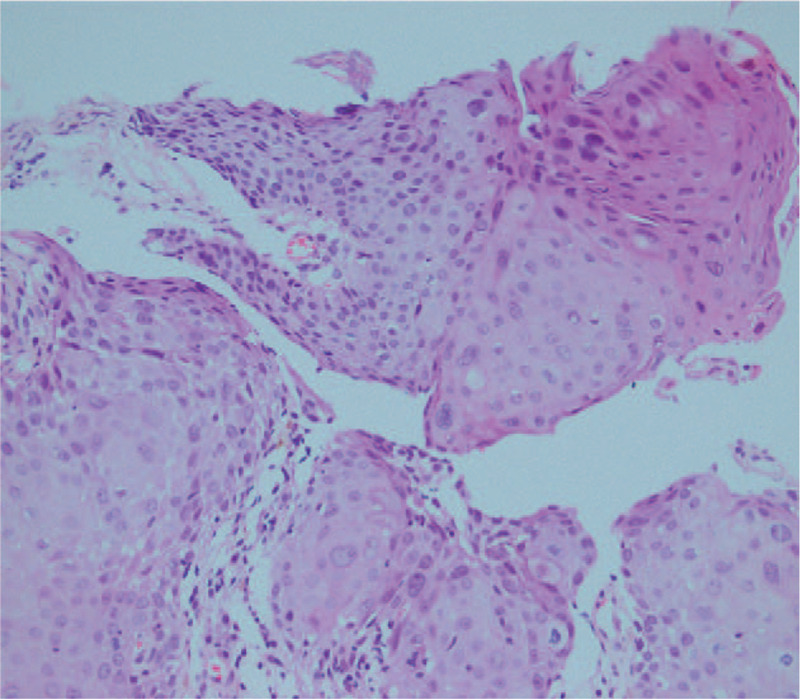
Histopathology examination of Perianal neoplasms (hematoxylin-eosin stain, ×10).

The histopathological findings of the stomach, colon, and small intestinal polyps were consistent, showing irregular, distorted, cystically dilated glands, lamina propria edema, multiple chronic inflammatory cell infiltration, chronic inflammatory cell infiltration, and eosinophilic granulocyte infiltration. After perianal tumor resection, pathology suggested that this was a perianal condylomatous lesion with malignant transformation, as well as well-differentiated squamous cell carcinoma. No tumor involvement was observed on the incisal margins (Fig. 6).

### 2.7 Final diagnosis

The patient presented with diffuse gastrointestinal polyposis, accompanied by alopecia, hyperpigmentation, and onychatrophia. All these symptoms were consistent with the diagnosis of CCS.

### 2.8 Treatment

Treatments included glucocorticoids (methylprednisolone 40 mg IV qd, which was gradually reduced to 30 mg PO), glutamine to protect the intestinal lining (3% alanyl glutamine 50 ml IV qd), and fat emulsion, amino acids, and glucose injection solution for nutritional support.

### 2.9 Outcome and follow-up

After about 3 weeks of treatment, the diarrhea and anorexia improved significantly, and the patient's body weight increased by 2.5 kg. At present, the patient has been followed up for 4 years. The patient was glucocorticoid-dependent: when the dose of methylprednisolone decreased to less than 20 mg/d, anorexia, diarrhea, and other clinical symptoms recurred. Thus, azathioprine (1.5 mg/kg/day) was added as a combination treatment and was effective in controlling the symptoms. At present, the hormone has been discontinued and azathioprine (1.0 mg/kg/day) continues as maintenance therapy. His body weight had increased by 13 kg (compared to the weight at hospitalization), and there has been no recurrence of the perianal lesions after resection.

## 3 Discussion

We here reported a case of CCS with perianal condyloma acuminatum, which had undergone malignant transformation; no such case had previously been reported. The main clinical manifestations in this case were diarrhea, anorexia, and emaciation. Examinations revealed gastrointestinal polyps, alopecia, cutaneous hyperpigmentation, and onychatrophia of the fingernails and toenails.^[2]^

A literature review aided in making the diagnosis of CCS. CCS is a rare disease without family clustering and with an unknown etiology. It is considered to be related to infection, autoimmunity, vitamin deficiency, mental stress, and fatigue, but the primary etiology is thought to be immune-related, for the following reasons. A 14-case CCS study from the Mayo Clinic showed that IgG4 was positive in 52% of CCS polyps, in 12% of juvenile polyps, and negative in normal controls, with statistically significant difference in prevalence (*P* < .01).^[3]^ Another study of 18 CCS cases from Peking Union Medical College Hospital drew similar conclusions.^[4]^ Moreover, some CCS cases may be complicated by Sjögren's syndrome, systemic lupus erythematosus, rheumatoid arthritis, scleroderma, and other autoimmune diseases.^[5]^ Most hormonal and immunosuppressive therapies are effective for CCS.^[3]^

Follow-up of the patient revealed that his wife did not have human papilloma virus infection, and the patient reported not having multiple sexual partners or homosexual intercourse; thus, the occurrence of condyloma acuminatum was considered to be an opportunistic infection. Condyloma acuminatum primarily occurs due to decreased immune clearance of human papilloma virus. CCS is also considered to be related to immune disorder, and thus, the occurrence of both conditions may be correlated. However, in the absence of reports of more cases, the exact relationship between the 2 diseases remains to be determined.

There are relatively few case reports on the characteristics of the small intestine in patients with CCS. The current case is the only one that demonstrates the characteristics of the small intestine with CCS disease through the combination of enteroscopy and capsule endoscopy. The characteristics of CCS polyps can be clearly observed by enteroscopy. In this case, polyps were also found to be distributed in the small intestine, especially in the duodenal, jejunal, proximal and terminal ileal regions. Capsule endoscopy, on the other hand, can clearly display the villi in the small intestine. We found that not only the villi in polyps were swollen and elongated, but also the villi in non-polyps, which was consistent with the findings of Heinzow et al and Wallenhors et al.^[6,7]^ Tomas et al and She et al showed that the mucosa between gastrointestinal polyps also had edema and that polyps tended to merge.^[8,9]^ These findings of the stomach, colon, and small intestine suggest that changes caused by CCS will not only lead to gastrointestinal polyps, but also be accompanied by changes in total digestive inflammation; this explains why the disease easily leads to clinical symptoms such as diarrhea, anorexia, and emaciation.

In this case, nutritional support and glucocorticoids were used as initial treatment. Due to repeated recurrence after glucocorticoid dose reduction, azathioprine (2 mg/kg/d) was used to control the disease and was effective. At present, the patient has maintained remission without obvious side effects. Current CCS treatments, used individually or in combination, include steroids, nutritional therapy, 5-aminosalicylate acid, histamine H2 receptor antagonists, anti-tumor necrosis factor α (TNF-α) agents, immunomodulators, and eradication of *Helicobacter pylori*.

Steroids are considered the mainstay of medical treatment, although the recommended dose and duration of their use have varied widely in the literature, with no current “gold standard”. The problem with hormone therapy is recurrence during dosage reduction, which occurs in nearly 40% of patients.^[3,10]^ Azathioprine, cyclosporin A, and anti-TNF agents can be used in relapsed patients,^[11–13]^ but these are mostly single cases or reported in small samples, and there is a lack of control studies on large samples.

For CCS patients with mild symptoms or early onset, 5-amino salicylic acid has also been reported to maintain long-term remission.^[14–15]^ Maintaining long-term remission is very important for CCS treatment, and can improve the quality of life of patients, effectively reduce the cancer rate, and effectively prolong the survival time.^[10]^ However, there is still no conclusion on the duration of maintenance treatment for this disease.

In conclusion, no CCS patient with perianal condyloma acuminatum with malignant transformation has been reported previously. Although the causal association of CCS and condyloma acuminatum in this case remains uncertain, clarification of the CCS etiology is possible through accumulation of similar cases. Through the observation of the small intestine by enteroscopy and capsule endoscopy, we found inflammatory changes throughout the small intestine, which were manifested as hyperemia and lengthening of the villi of the small intestine, with a seaweed-like appearance. This case can help deepen our understanding of CCS and improve the diagnosis and treatment of the disease, by emphasizing the role of whole digestive tract endoscopy of CCS patients.

## Acknowledgments

We would like to thank Editage (www.editage.cn) for English language editing. Thanks Ying Wang for the pictures revision and Thanks Da-hua Zhao for his help in histopathology.

## Author contributions

**Conceptualization:** Wei Wang.

**Methodology:** Wei Wang, Xian-yong Cheng, Feng Xue.

**Writing – original draft:** Wei Wang, Hai-yan Liu, Hai-feng Lian.

**Writing – review & editing:** Cheng-xia Liu.
